# Telerehabilitation: Review of the State-of-the-Art and Areas of Application

**DOI:** 10.2196/rehab.7511

**Published:** 2017-07-21

**Authors:** Alessandro Peretti, Francesco Amenta, Seyed Khosrow Tayebati, Giulio Nittari, Syed Sarosh Mahdi

**Affiliations:** ^1^ School of Pharmacy Telemedicine and Telepharmacy University of Camerino Camerino Italy

**Keywords:** telerehabilitation, rehabilitation, telemedicine, health care, remote rehabilitation assistance

## Abstract

**Background:**

Telemedicine applications have been increasing due to the development of new computer science technologies and of more advanced telemedical devices. Various types of telerehabilitation treatments and their relative intensities and duration have been reported.

**Objective:**

The objective of this review is to provide a detailed overview of the rehabilitation techniques for remote sites (telerehabilitation) and their fields of application, with analysis of the benefits and the drawbacks related to use. We discuss future applications of telerehabilitation techniques with an emphasis on the development of high-tech devices, and on which new tools and applications can be used in the future.

**Methods:**

We retrieved relevant information and data on telerehabilitation from books, articles and online materials using the Medical Subject Headings (MeSH) “telerehabilitation,” “telemedicine,” and “rehabilitation,” as well as “disabling pathologies.”

**Results:**

Telerehabilitation can be considered as a branch of telemedicine. Although this field is considerably new, its use has rapidly grown in developed countries. In general, telerehabilitation reduces the costs of both health care providers and patients compared with traditional inpatient or person-to-person rehabilitation. Furthermore, patients who live in remote places, where traditional rehabilitation services may not be easily accessible, can benefit from this technology. However, certain disadvantages of telerehabilitation, including skepticism on the part of patients due to remote interaction with their physicians or rehabilitators, should not be underestimated.

**Conclusions:**

This review evaluated different application fields of telerehabilitation, highlighting its benefits and drawbacks. This study may be a starting point for improving approaches and devices for telerehabilitation. In this context, patients’ feedback may be important to adapt rehabilitation techniques and approaches to their needs, which would subsequently help to improve the quality of rehabilitation in the future. The need for proper training and education of people involved in this new and emerging form of intervention for more effective treatment can’t be overstated.

## Introduction

In the last few years, telemedicine applications have been increasing due to the development of new computer science technologies and of more advanced telemedical devices. Long-distance communication can be easily achieved by videoconferencing, email, and texting, to name a few. Today there is the possibility of controlling robots, robotic arms, or drones at a distance. Thanks to these advancements, the course of human action has been considerably transformed [1]. During the last 20 years, demographic changes and increased budget allocation in public health have improved new rehabilitative practices [2]. Rehabilitation is an old branch of medicine, but in the last few years, new telecommunication-based practices have been developed all over the world. These particular approaches in the field of rehabilitation are commonly defined as telerehabilitation, which should be considered as a telemedicine subfield consisting of a system to control rehabilitation at a distance [3].

Telerehabilitation has been developed to take care of inpatients, transferring them home after the acute phase of a disease to reduce patient hospitalization times and costs to both patients and health care providers. Telerehabilitation allows for treatment of the acute phase of diseases by substituting the traditional face-to-face approach in the patient-rehabilitator interaction [4]. Finally, it can cover situations in which it is complicated for patients to reach traditional rehabilitation infrastructures located far away from where they live.

Controlled studies on rehabilitation have demonstrated that quick management of an injury or a disease is critical to achieve satisfactory results in terms of increasing a patient’s self-efficacy. Hence, a rehabilitation program should start as soon as possible, be as intensive as possible, be prolonged, and continue during the recovery phase. A major factor is the initiation time, which, in general, should begin as soon as possible. In most cases, the initial stages of rehabilitation, after the occurrence of a disease or injury, could be performed by patients at home even if they need accurate and intensive treatment. For these reasons, telerehabilitation was developed to achieve the same results as would be achieved by the normal rehabilitation process at a hospital or face to face with a physiotherapist. Various types of telerehabilitation treatments and their relative intensities and duration have been reported [5].

**Figure 1:**
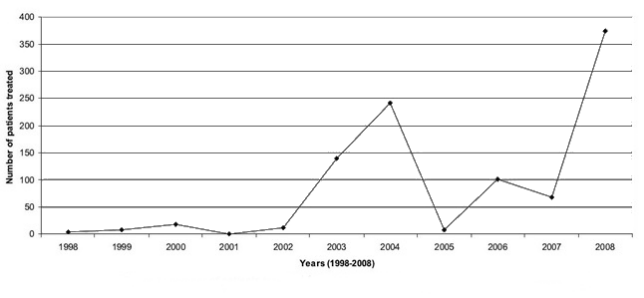
Number of patients treated from 1998 to 2008 through telerehabilitation techniques.

The first scientific publication on telerehabilitation is dated 1998 and, in the last few years, the number of articles on the topic has increased, probably because of the emerging needs of people and due to the development of exciting new communication and computer technologies. Figure 1 shows the number of patients treated through telerehabilitation from 1998 to 2008 according to studies published in the international literature [2].

A remarkable increase in the number of patients treated by telerehabilitation is noticeable from 2002 to 2004. After a subsequent decrease, the number of patients assisted by telerehabilitation increased starting from 2007, probably due to the support of new technologies and the overcoming of the initial skepticism to which every new technology is subjected.

Telerehabilitation is primarily applied to physiotherapy [6,7], and neural rehabilitation is used for monitoring the rehabilitative progress of stroke patients [8]. Telerehabilitation techniques mimic virtual reality [9-12] and rehabilitation for neurological conditions by using robotics and gaming techniques [13]. Quite often, telerehabilitation has been associated with other nonrehabilitative technologies such as remote monitoring of cardiovascular parameters, including electrocardiogram (ECG), blood pressure, and oxygen saturation in patients with chronic diseases [14]. These technologies belong to another telemedicine branch called telemonitoring, which has been widely developed and used in recent years. A few studies were also centered on the economic aspects of the use of telerehabilitation to reduce the costs of hospitalization [15]. We reviewed the status and future perspectives of telerehabilitation by analyzing their impact on patients’ everyday life. The main topics taken into account were (1) the status of telerehabilitation and analysis of the main medical specialties where it is being applied, (2) quality-of-life improvement due to telerehabilitation, and (3) the future of telerehabilitation.

**Table 1:** Characteristics of studies on telerehabilitation reviewed.

First author, date, reference	Type of article	Rehabilitation area	Sample size	Article key points	Positive aspects	Barriers and limitations
Ackerman, 2010 [1]	Original research	Multiple rehabilitation areas	Literature review	Next-generation telehealth tools	Devices are available at home; electronic health record available for each person; interaction of multiple systems.	People and technological systems are not ready (data flow and incompatibility between telerehabilitation systems).
Rogante, 2010 [2]	Review article	Multiple rehabilitation areas	Literature review	Overview of telerehabilitation literature	Provides some techniques at a distance.	Health care providers are not ready; comprehensive studies are lacking.
Zampolini, 2008 [3]	Review article	Multiple rehabilitation areas	Literature review	Overview of telerehabilitation literature and a study	The possibilities of using telerehabilitation as standard in the future.	Technologies, patients, and health care providers are not ready.
Carey, 2007 [4]	Original research	Physiotherapy	Literature review	Cortical reorganization after stroke	Telerehabilitation may be effective in improving performance in patients with chronic stroke.	No clear advantage produced over the same amount of practice of random movements.
Parmanto, 2008 [5]	Review article	Multiple rehabilitation areas	Literature review	Telerehabilitation from informatics perspective	Information technology and telerehabilitation are the future.	Health care providers are not ready to manage an everyday telerehabilitation approach.
Mani, 2016 [6]	Review article	Physiotherapy	Literature review	Telerehabilitation in musculoskeletal disorders	Telerehabilitation-based physiotherapy assessment is technically feasible.	Telerehabilitation-based physiotherapy assessment was not feasible or reliable for lumbar spine posture, orthopedic special tests, neurodynamic tests, and scar assessment.
Gal, 2015 [7]	Original research	Physiotherapy	Literature review	Kinect-based system in physiotherapy	Kinect can greatly help people in rehabilitation.	Not present.
Jagos, 2015 [8]	Clinical trial	Cardiac rehabilitation	5 patients	Rehabilitation after stroke	The system used could be used for further analysis.	Not present.
Keshner, 2007 [9]	Original research	Multiple rehabilitation areas	Literature review	VR^a^as a treatment intervention	VR should be used more in the future.	People are not ready.
Larson, 2014 [10]	Review article	Multiple rehabilitation areas	Literature review	VR treatment	VR is effectively used for telerehabilitation.	Further studies are needed to optimize the techniques.
Kenyon, 2004 [11]	Review article	Multiple rehabilitation areas	Literature review	VR treatment	The virtual environment can be a valuable tool for therapeutic interventions that require adaptation to complex, multimodal environments.	Not present.
Lewis, 1997 [12]	Review article	Multiple rehabilitation areas	Literature review	VR treatment and human factors	VR has many potentialities in health care.	Some users experienced adverse effects during and after exposure to VR environments (ocular problems, disorientation and balance disturbances, and nausea).
Burdea, 2013 [13]	Case study	Physiotherapy and neurological rehabilitation	Literature review	Cerebral palsy motor control improvement	Game-based robotic training of the ankle benefits gait in children with cerebral palsy.	Additional studies are needed to quantify the level of benefit and for comparing different approaches.
Busch, 2009 [14]	Clinical trial	Cardiac rehabilitation	4 patients	Electrocardiography, blood pressure, and oxygen saturation in cardiac patients	The system shown is acceptable.	Electrocardiogram connection (27%) and blood pressure reading problems (23%); more reliability is needed.
Dinesen, 2012 [15]	Case study	Multiple rehabilitation areas	60 patients	Telehealth in pulmonary disease patients	Not present.	Future work requires large-scale studies of prolonged home monitoring with more extended follow-up.
Giansanti, 2013 [18]	Original research	Multiple rehabilitation areas	Literature review	Validation of a portable care system	Very low costs compared with optoelectronic solutions and other portable solutions; very high accuracy, also for patients with imbalance problems; good compatibility with any rehabilitative tool.	Not present.
Kairy, 2016 [19]	Clinical trial	Physiotherapy	104 patients	Upper limb through VR	This approach can enhance continuity of care once patients are discharged from rehabilitation.	Not present.
Myers, 2003 [20]	Original research	Cardiac rehabilitation	Literature review	Cardiology overview	Not present.	Not present.
Piotrowicz, 2012 [21]	Clinical trial	Cardiac rehabilitation	75 patients	Home-based cardiac rehabilitation in heart failure patients	The system used is reliable.	Further studies are required.
Fletcher, 2001 [22]	Original research	Cardiac rehabilitation	Literature review	Exercise standards for testing and training	Not present.	Not present.
Piotrowicz, 2010 [23]	Clinical trial	Cardiac rehabilitation	152 patients	Home-based telemonitoring system	The system is effective and usable.	Not present.
Rose, 2005 [24]	Review article	Neurological rehabilitation	Literature review	VR in brain damage	VR has the potential to assist current rehabilitation techniques and will be an integral part of cognitive assessment and rehabilitation in the future.	Brain damage rehabilitation is still a relatively undeveloped field.
Satava, 1995 [25]	Original research	Multiple rehabilitation areas	Literature review	VR	Not present.	Not present.
Rizzo, 2005 [26]	Original research	Neurological rehabilitation	Literature review	VR, brain, and therapy	VR has many potentialities in medicine.	VR rehabilitation is still in an early phase of development characterized by successful proof of concept.
Linder, 2015 [27]	Clinical Trial	Neurological rehabilitation	99 patients	Improving quality of life and depression after stroke	A robot-assisted intervention may be a valuable approach for improving quality of life.	Not present.
Holst, 2017 [28]	Original research	Neurological rehabilitation	Literature review	Depression treatment	Internet-mediated cognitive behavioral therapy is an attractive alternative for some, but not all, patients with depression in primary care.	Lack of face-to-face meeting and human contact.
Vaughan, 2016 [29]	Review article	Neurological rehabilitation	Literature review	State-of-the-art of VR	VR could be used to treat neurological patients.	Not present.

^a^VR: virtual reality.

## Methods

### Data Sources and Study Selection

We systematically searched the literature in the PubMed and Medline databases, *British Medical Journal*, Oxford Journals, Biomed Central, and CINAHL using the Medical Subject Headings (MeSH) “telerehabilitation,” “telemedicine,” and “rehabilitation,” as well as “disabling pathologies” *.* Parameters applied were English language, at least one keyword corresponding to the search terms in the title or abstract, and study based on the evaluation of clinical trials. An additional evaluation criterion was the publication of articles in peer-reviewed journals. The search was carried out in 2016 for the years January 1996 to January 2016. Moreover, we selected and examined 45 books and other online materials through Google search, university of Camerino E-database, and the central library of University of Camerino. We retrieved more than 400 articles on telerehabilitation or related topics. A further analysis performed by 2 researchers independently reading article titles and abstracts reduced the results to less than the 25% of articles.

### Exclusion Criteria

We excluded studies or other materials published before 1996 from our analysis. This is because, as Figure 1 shows, the first effective telerehabilitation procedures started in 1998. Therefore, the selected articles were published between 1996 and 2016. We also excluded articles published in nonpeer-reviewed journals, as well as pilot studies, due to the small number of patients investigated. Only English-language articles were selected. Finally, we discarded articles without the terms telerehabilitation, disabling pathologies, telemedicine, or rehabilitation in the title or keywords.

### Quality Assessment

We evaluated the relevant articles with the standard criteria of the Newcastle-Ottawa Scale for assessing the quality of nonrandomized studies in meta-analyses [16]. Overall study quality was defined as poor (score 0-4), moderate (5-6), or good (7-9). The score was based on the following filters that could be attributed to a review article: comparability, and desired outcome. In addition, we analyzed various parameters of each article. The scores depended on these parameters.

**Figure 2:**
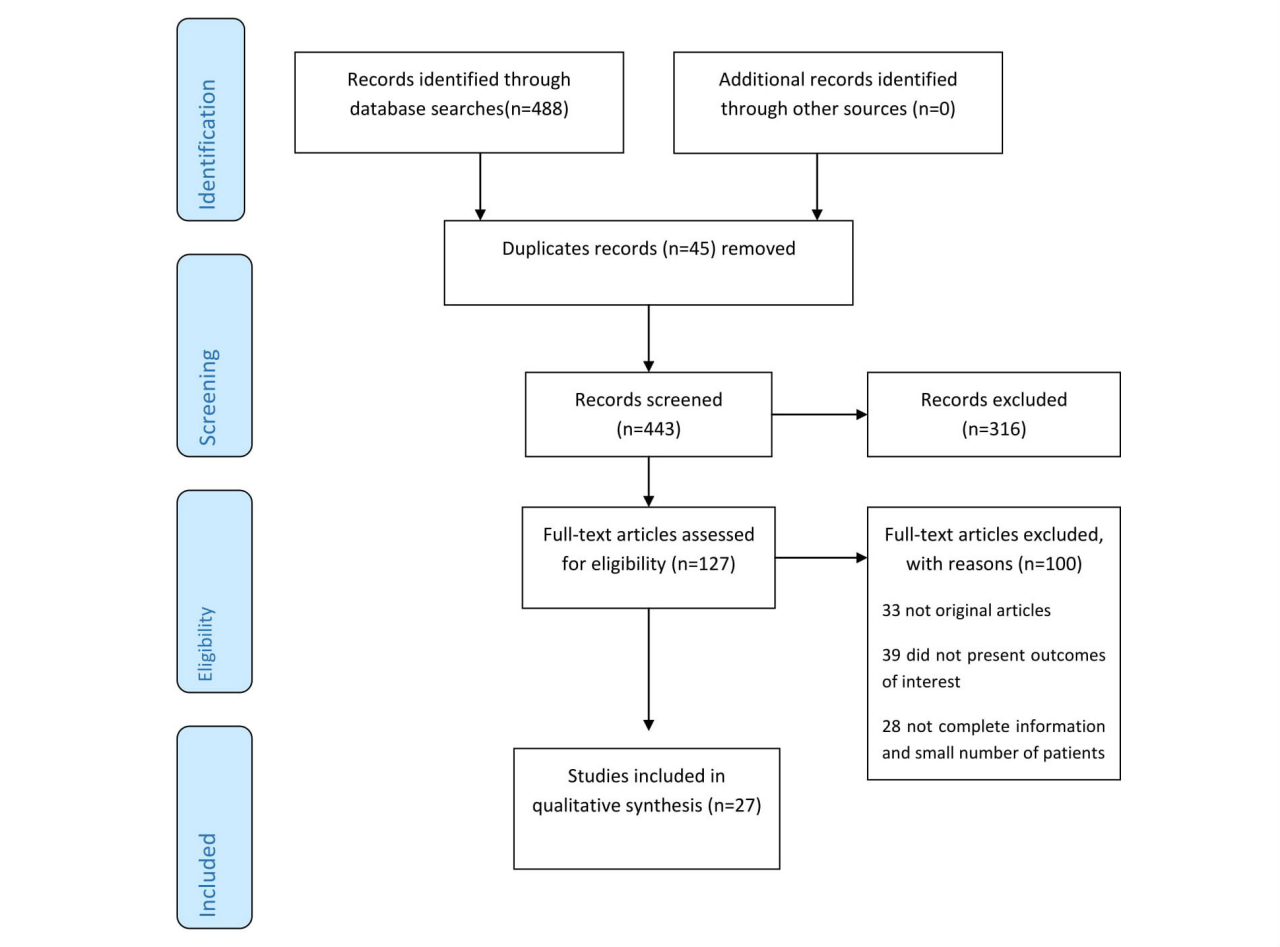
Preferred Reporting Items for Systematic Reviews and Meta-Analyses (PRISMA) flowchart.

## Results

### Evaluation Outcomes

The literature search identified 488 abstracts, 127 of which we analyzed in detail. Among these 127 articles, we excluded 100 in a full-text analysis (Figure 2 [17]). The search analysis showed that, although all these articles matched with the keywords we used, most of them were pilot studies evaluating the response of the system in the real environment in a small sample group of patients. We did not discard these articles because pilot studies are also a valuable source of information, but we considered them as an experience in relation to the patients involved. Finally, we chose only a few of articles (n=27) to assess applications of telerehabilitation to meet patients’ needs in everyday life [18,19].

From our literature analysis, we identified that telerehabilitation was used primarily in cardiac, neurological, and physiotherapy rehabilitation. Table 1 summarizes data derived from the literature and supplemented by additional information when available [1-15,18-29].

### Cardiac Telerehabilitation

In chronic cardiac diseases, rehabilitation is one of the main tools used to improve the quality of life of patients, along with a drastic reduction of cardiac risk factors, mainly through lifestyle changes. Inpatient rehabilitation is in general effective and efficient, whereas in outpatients the quality of rehabilitation is limited. Only 13% to 40% of the total cardiac patient population in Germany performed cardiac rehabilitation in a supervised and controlled-phase program [14]. Some studies showed that at least 5 to 30 minutes of aerobic sessions per week reduce cardiac risk factors [20]. Patients do not join supervised and controlled-phase rehabilitation programs due to scheduling conflicts, difficulties in reaching the training phase, and a reluctance to perform the exercise in a group. Another study showed that home-based telemonitored cardiac rehabilitation (HTCR) is a new method of rehabilitation for stable heart failure patients [21]. For at-home exercise, transtelephonic ECG could be a good substitute for outpatient visits [22] and probably HTCR produces quality-of-life improvements similar to those obtained by standard outpatient-based cardiac rehabilitation [23].

An example of telerehabilitation applied to cardiovascular diseases is the SAPHIRE system [14]. It consists of a bicycle with a touch screen and wireless sensors to check the patients’ ECG, blood pressure, and oxygen saturation in real time. At the hospital, the supervising staff can connect remotely to the patient’s computer touch screen to customize the exercises based on the results of a previous exercise stress test. They can monitor the patient’s health condition in real time during the rehabilitation, and they can stop the exercises if abnormal sensors values are detected. In particular, the system has three different training forms: constant load, intervals, and heart rate control. If any limits are exceeded, the patient is alerted through an icon to reduce the load or to immediately abort the exercises. The SAPHIRE system was used in 4 patients with 13 staff members. During the experimental phase, no serious events related to heart disease occurred, but some difficulties were observed regarding sensor operation. In the 39 training sessions completed, in 27% of them the ECG connection could not be established and in 23% blood pressure measurement failed.

The more important advantage of this kind of telerehabilitation system is common to other telehealth systems. Patients can follow their rehabilitation program at a distance (eg, at home) saving time and money, and avoiding unnecessary travel and discomfort to the patient. The disadvantages are also common among different telerehabilitation systems. These include limited flexibility in the use of the various medical devices appropriate to patients’ differing needs.

### Neurological Telerehabilitation

In the case of neurological diseases such as brain injury or cognitive problems, the best rehabilitation for patients is to stimulate the brain with adequate environmental interactions. Probably due to the short history of neurological rehabilitation techniques, neurological telerehabilitation approaches are not clearly defined at this point and have no concrete theoretical bases [24]. Recent research in human-computer interfaces has improved the effectiveness of virtual reality. Virtual reality consists of simulations through dedicated machines such as personal computers with specific graphical features of a real environment. The machine could be interfaced with devices such as robotic arms, robotic legs, data gloves, and smart glasses. Such smart devices can be used in a 3-dimensional environment simulation, and they can allow for a greater sense of immersion in the virtual environment [10].

The first conference on virtual reality applied to medicine was the Medicine Meets Virtual Reality conference [25]. Advantages of this new type of approach over standard care were discussed. The positive results encouraged the use of this smart technology. Performing exercises inside a laboratory (ie, in front of a computer) avoids the unnecessary risk that comes with performing the same exercises in a real and dangerous environment, can establish the simulated environment in relation to the patient’s condition, and optimizes the difficulty of the environment according to the patient’s neurological severity [26].

Health care treatment closer to the needs of the specific pathologies of the patient improves the quality of life and often decreases the duration of treatment. It has been shown that virtual reality can be used for the assessment and rehabilitation of specific disabilities resulting from brain injury, executive dysfunction, memory impairments, spatial disability, attention deficits, and unilateral visual neglect. A virtual urban environment for the treatment of 27 patients with moderate and severe brain injuries was developed in which patients needed to navigate in the simulator. However, this study showed no improvement due to the number of repetitions [24].

Another example of a telerehabilitation system is the Rehab@Home framework used to perform rehabilitation in the domestic setting for stroke patients [8]. The framework consists of instrumented insoles connected wirelessly to a third-generation tablet computer, a server, and a graphic Web interface for medical experts. Rehabilitation progress is automatically analyzed after assessment tests are executed in the tablet computer. Both the systems (Rehab@Home, virtual urban environment) were accepted by patients and doctors because of good results obtained. Perhaps the systems described above will not be used widely in the future, but they will contribute to improve these approaches in the future, with better cardiac telerehabilitation applications.

A telerehabilitation system was applied to 99 poststroke patients to evaluate their quality of life. The authors observed a statistically significant change in both interventions (normal and robotic rehabilitations). Actually, both modalities were effective in improving quality of life and depression outcomes for participants at less than 6 months after their stroke. The goal of this study was to obtain better results for robotic rehabilitation, but the findings obtained did not show significant differences between the 2 groups [27].

A telerehabilitation project called H-CAD was developed from 2003 to 2005. H-CAD is a system for patients with multiple sclerosis, stroke, or traumatic brain injury for performing upper limb rehabilitation treatment, at home. A help desk was developed to guide the patients in developing a proper exercise regimen by evaluating the performance periodically. Patients had the possibility to interact with doctors at the hospital through a teleconferencing system. The process was carried out in 2 test phases. The first phase was to test the results of the system with volunteers inside a hospital. The second phase was to test the system at home with ad hoc patients. The results were encouraging, and the doctors observed a marked improvement in patients using this system [3].

More recently, the use of a telerehabilitation approach in the management of patients with depression was studied [28]. Here, the authors used an Internet-mediated cognitive behavioral therapy (iCBT) system to treat depression remotely. Unfortunately, considering that depression is an important, modern, and widespread psychiatric disorder, these results were not conclusive [28]. Eventually an individual treatment design seems to be preferred, and elements of iCBT could be included as a complement when treating depression in primary care. These procedures may be economically important because they could relieve the overall treatment burden of depression.

### Physiotherapy Applied to Telerehabilitation

Musculoskeletal disorders have a high impact on health care provision. A controlled study was conducted to assess the effectiveness of a telerehabilitation approach instead of standard face-to-face practice. A literature review analyzed 898 studies on the validity and reliability of Internet-based physiotherapy assessment for musculoskeletal disorders. Most of the telerehabilitation approaches were valid if they were applied for some physical diseases, except for lumbar spine posture, where the final score was not conclusive. In fact, results showed that the intervention had effectiveness scores from low to moderate [6].

Another study demonstrated the use of Microsoft Kinect, a motion-sensing input device, to detect patients’ posture and movement, and it enabled caregivers to develop custom exercise patterns for each patient. Tests confirmed that the intervention had several benefits, particularly in creating a customized physical exercise program for physical rehabilitation [7].

## Discussion

The application of telemedicine to cardiology, neurology, and rehabilitation is growing fast. For instance, its use in neurology in emergency departments is particularly critical because so many of them do not have a full-time neurologist. In 2016 it was reported that about 125,000 patients who had a stroke or symptoms of stroke used telemedicine-based technology in one form or another during treatment or rehabilitation. Telerehabilitation is a young field of telemedicine (Figure 1), and it may cover different areas of medicine [2]. As a new field, it is still undergoing research and development, and all the applications available are being tested with only a limited number of patients (Table 1). Every system analyzed is a basic one used to check the effectiveness and the responsiveness of patients and doctors to this new approach. It is easily observable that the technology is ready to be used for telerehabilitation. With the support of wireless sensors, microcomputers, and communications systems, it is possible to develop a telerehabilitation system, but further research is required to determine the effectiveness of these systems.

### Advantages and Disadvantages

Like every technology, telerehabilitation has some advantages and disadvantages. In terms of advantages, home telerehabilitation systems are cost effective if the intervention is just used to monitor or evaluate patients during corrective therapy [14,29]. The possibility to stay in touch with telematic technologies allows patients with serious pathologies, such as severe cognitive deficits, to perform physiotherapy at home without having to make tiring journeys. In terms of disadvantages, a problem could be the loss of human contact (face-to-face interaction) with the doctor. Moreover, for each patient, system operators are required to optimize the teletherapy according to the type of disease, and sometimes this is not possible due to high costs.

### Conclusion

This review evaluated different application fields of telerehabilitation, highlighting its benefits and drawbacks. In conclusion, this analysis has shown that telerehabilitation is a new and interesting field but, unfortunately at present, there are no standard procedures or protocols, and different telerehabilitation facilities are being used for pilot studies only. Herein, we suggest the need for further research to improve the electronic equipment and devices, and to make their application as flexible as possible. This approach should significantly increase the reliability and effectiveness of telerehabilitation equipment to treat specific patient problems. Furthermore, in this context, feedback from patients may be important to update rehabilitation techniques to improve the quality of the rehabilitation itself. On the other hand, an important aspect of the future success of telerehabilitation involves proper training of people involved in these new forms of intervention, which may lead to more effective rehabilitation.

## Abbreviations

ECG: electrocardiogram
HTCR: home-based telemonitored cardiac rehabilitation
iCBT: Internet-mediated cognitive behavioral therapy
MeSH: Medical Subject Headings
